# Public Health Approaches to Perinatal Substance Use: An Overview of Strategic Directions

**DOI:** 10.1007/s10995-023-03792-4

**Published:** 2023-11-06

**Authors:** Amani Echols, Stacy Collins, Sanaa Akbarali, Ramya Dronamraju, Keriann Uesugi

**Affiliations:** 1 Association of Maternal & Child Health Programs, Washington DC, USA; 2 Association of State and Territorial Health Officials, Arlington, VA USA; 3 Maternal and Child Health Bureau, Health Resources and Services Administration, U.S. Department of Health and Human Services, Rockville, MD USA

Almost one in four pregnancy-related deaths is attributed to mental health conditions, including substance use disorder (SUD), making them the leading underlying cause of pregnancy-related deaths, according to data from 2017 to 2019 in 36 states (Trost et al., 2022). Between 2017 and 2020, 1249 pregnancy-associated deaths, most occurring in the late postpartum period, were overdose-related (Bruzelius & Martins, 2022). Rising rates of overdose deaths, fueled by the COVID-19 pandemic, have exacerbated concerns about perinatal SUD (Lien et al., 2023; Spencer et al., 2022; United States Government Accountability Office, 2022). Now is a critical time to invest in the health and wellness of perinatal people with SUD and their families.

This special Maternal and Child Health Journal issue builds on the evidence base, featuring articles that share the latest research, program, and policy initiatives in perinatal SUD. While the impact of prenatal substance exposure on the health and development of infants has been written about extensively, this collection of articles offers a more explicit focus on the health of pregnant and postpartum people who use substances. Moreover, the articles in this issue highlight tangible program and policy approaches to preventing and mitigating the harms of perinatal substance use and improving maternal and child health.

The Association of Maternal & Child Health Programs (AMCHP) and the Association of State and Territorial Health Officials (ASTHO) sponsored the special issue, with funding from the Maternal and Child Health Bureau within the U.S. Health Resources and Services Administration (HRSA-MCHB). AMCHP and ASTHO have engaged states advancing public health strategies to address perinatal SUD through the PRISM (Promoting Innovation in State and Territorial MCH Policymaking) project. In their article, Akbarali and coauthors describe the PRISM national learning community and the priorities of the participating jurisdictions. This special issue is a capstone of the PRISM project.

## Strategic Directions Identified by The Authors

### Assess the State of Perinatal Behavioral Health

Several articles define the scope of perinatal SUD, identify underserved populations, and reveal gaps and inefficiencies in our current behavioral health care system. Setting the stage, West and coauthors estimate that between 2016 and 2020, the national neonatal abstinence syndrome rate declined by 20%, while the estimated national rate of prenatal substance exposure experienced an 11% increase. The authors hypothesize that increased use of substances other than opioids (e.g., cannabis, nicotine, alcohol, and non-opioid illicit drugs) influenced this trend.

Using data from the New Mexico Maternal Mortality Review Committee, Fuchs and coauthors find that most perinatal SUD-related deaths in New Mexico involve polysubstance use, and that perinatal people with SUD-related deaths were more likely to die six weeks to 12 months postpartum, compared to perinatal people with non-SUD-related deaths.

Two articles, authored by Elmore, Decker, and their respective coauthors, question the utility and accuracy of hospital administrative data for identifying maternal opioid use at delivery and introduce the promise of statewide perinatal substance use and neonatal abstinence syndrome surveillance.

### Build a Diverse and Culturally Competent Perinatal Behavioral Health Workforce

Much of the conversation on mental and behavioral health focuses on strengthening the system's capacity to meet the demand for treatment because more than one-third of Americans live in a designated Mental Health Professional Shortage Area (HRSA, 2023), and the pool of providers specializing in perinatal mental health is critically low. Articles by Reese, Sternberger, Haerizadeh-Yazdi, and their respective coauthors demonstrate the importance of investing in care coordination, peer support, and doula care to expand the perinatal behavioral health workforce. The authors stress that provider training on stigma reduction and cultural competency are essential components of workforce capacity building.

### Integrate Perinatal and Behavioral Health Care Services

In addition to severe provider shortages, the siloing of perinatal and behavioral health care makes accessing and navigating the system of care challenging. However, integrated care aims to eliminate access barriers and meet people where they are through a no-wrong-door approach [Substance Abuse and Mental Health Services Administration (SAMHSA), 2022]. Preventive screening is a foundational approach to integrated care. Reese and coauthors demonstrate the feasibility of using telehealth to administer screening, brief intervention, and referral to treatment (SBIRT) in an outpatient obstetric setting. Three additional articles demonstrate opportunities to co-locate care. First, Sternberger and coauthors describe how the Moms Do Care program in Massachusetts established and expanded 11 co-located multidisciplinary medical and behavioral health care teams to care for pregnant and parenting people with SUD and their families. Second, Goyal and coauthors discuss incorporating maternal opioid use disorder treatment within the group well-child care model. Third, Cheedalla and coauthors explain the benefits of a linkage-to-care model involving co-located obstetrical care and SUD treatment for perinatal people with SUD and Hepatitis C.

### Advance Nonpunitive and Harm Reduction Approaches

The harmful effects of criminalizing pregnant and parenting people who use substances stem from long-standing, inequitable drug policies that disproportionately impact Black and Brown communities (Fornili, 2018; Newkirk II, 2017). Pregnant people who use substances experience additional stigma and surveillance compared to non-pregnant people who use substances because their substance use has the potential to impact the fetus, calling into question their maternal fitness and often leading to punitive responses (Terplan et al., 2015; Volkow, 2023). According to Reddy and coauthors, infants with documented prenatal substance exposure experience an accelerated timeline of child protective services (CPS) involvement compared to infants without documented prenatal substance exposure. To protect the maternal-infant dyad, Work and coauthors call for uncoupling medical decisions from CPS reporting and involvement. Sharp and coauthors share how New Mexico achieved this by implementing a state policy that expands the federal Comprehensive Addition and Recovery Act of 2016 to incorporate nonpunitive approaches to care.

Perinatal harm reduction is a powerful paradigm that deconstructs the stigmatizing narratives about pregnant and parenting people who use substances and employs evidence-based interventions to reduce the harms associated with substance use (SAMHSA, 2023; National Harm Reduction Coalition & Academy of Perinatal Harm Reduction, 2022). Joelle Puccio explains that harm reduction is rooted in a human rights and reproductive justice framework and allows people who use substances to seek help without experiencing punitive responses.

### Facilitate Change by Strengthening Systems of Care for Perinatal People with SUD

The interrelated challenges contributing to perinatal SUD call for multistakeholder coordination to build a system of care that offers comprehensive services and support. Through an innovative multistakeholder group modeling workshop, Simon and coauthors demonstrate the value of adopting a systems-strengthening approach to support perinatal people with SUD.

Strengthened systems of care can link medical and nonmedical sectors connected to the social determinants of health (SDoH). For instance, Wingo and coauthors highlight that women with SUD who are experiencing homelessness have limited access to reproductive health services. Furthermore, lack of access to the SDoH is connected to stressful live events, histories of trauma, and adverse childhood experiences (ACEs). In their research, Duka and coauthors find that half of the birthing people referred to CPS for maternal substance use have four or more ACEs, including ACEs related to household challenges (e.g., parental separation/divorce), raising concerns about intergenerational trauma. Yakubu and coauthors examine whether perinatal people from communities of color who use substances are at greater risk of experiencing stressful life events, such as economic or housing instability, due to racism and other longstanding systemic factors. The authors advise universal screening of the SDoH needs and trauma histories of perinatal people with SUD.

Broadening the scope of care to encompass the SDoH can minimize risk factors and promote health and wellness across the life course. As Kimá Taylor suggests in her article, a new perinatal behavioral health system must center wellness and people impacted by perinatal SUD. Equitable access to and outcomes from a full continuum of resources are needed to improve the health of individuals and families.

## The Way Forward

Improving outcomes for perinatal people with SUD requires collective action. The strategic directions featured in this special issue offer promising program and policy options for state and local health officials, public health and MCH practitioners, health care professionals, and policymakers to consider. The recommendations in this special issue reinforce and strengthen the evidence base, yet opportunities remain to address the root causes of perinatal SUD and create a sustainable continuum of care for those affected by it. An expansive approach to structural change must be embraced in partnership with people with perinatal SUD experience.

In tandem with the strategic directions presented, the way forward must resolutely connect often siloed topic areas, such as:

- Perinatal physical and mental health services
- Mental health and substance use disorders
- Maternal, infant, and family health
- Perinatal SUD and access to SDoH and reproductive justice.

Due in large part to federal, national, and jurisdictional financial and strategic priorities, the supplement primarily focuses on preliminary program evaluations and screening and treatment of opioid use during pregnancy. However, the authors recognize the impact of alcohol, tobacco, and cannabis use on maternal and infant health outcomes. Future research and funding should focus on polysubstance use during the prenatal and postpartum periods and identify opportunities to provide comprehensive care and treatment for these individuals. Increased funding is also needed to perform more rigorous program and policy evaluations. Alongside concerted efforts from public funders, Scott and coauthors recommend that the philanthropic community invest in perinatal SUD innovations that build capacity and partnerships to achieve sustainable and high impact interventions.

The authors hope this special issue will generate new lines of inquiry and scale up evidence-based programs and policies to improve the lives of people with perinatal SUD and their families. This special issue continues the steady call for public health leaders and stakeholders to address perinatal SUD through a compassionate, holistic wellness lens for birthing people and families.

## Data Availability

N/A.

## Code Availability

N/A.
