# Correction: Public Health Approaches to Perinatal Substance Use: An Overview of Strategic Directions

**DOI:** 10.1007/s10995-023-03869-0

**Published:** 2023-12-26

**Authors:** Amani Echols, Stacy Collins, Sanaa Akbarali, Ramya Dronamraju, Keriann Uesugi

**Affiliations:** 1 Association of Maternal & Child Health Programs, Washington, DC USA; 2 Association of State and Territorial Health Officials, Arlington, VA USA; 3 Maternal and Child Health Bureau, Health Resources and Services Administration, U.S. Department of Health and Human Services, Rockville, MD USA

**Correction to: Maternal and Child Health Journal**

10.1007/s10995-023-03792-4

The original version of this article unfortunately contained a typo in the text.

In Build a Diverse and Culturally Competent Perinatal Behavioral Health Workforce section, the author’s name Yazdi in the sentence “Articles by Reese, Sternberger, Yazdi, and their…” would have been published as Haerizadeh-Yazdi. So, the correct sentence should read as “Articles by Reese, Sternberger, Haerizadeh-Yazdi, and their…”.

In Facilitate Change by Strengthening Systems of Care for Perinatal People with SUD section, the author’s name Esernio-Jenssen in the sentence “In their research, Esernio-Jenssen and coauthors find that half…” would have been published as Duka. So, the correct sentence should read as “In their research, Duka and coauthors find that half…”.

The original article has been corrected.

